# Supplementary material for: Using Transfer Learning Method to Develop an Artificial Intelligence Assisted Triaging for Endotracheal Tube Position on Chest X-ray
Source: Diagnostics (Basel). 2021 Oct 6;11(10):1844. doi: 10.3390/diagnostics11101844 (PMC8534985; doi:10.3390/diagnostics11101844)
Supplement: Supplementary file 1 [file diagnostics-11-01844-s001.zip › diagnostics-1369857-supplementary.pdf]

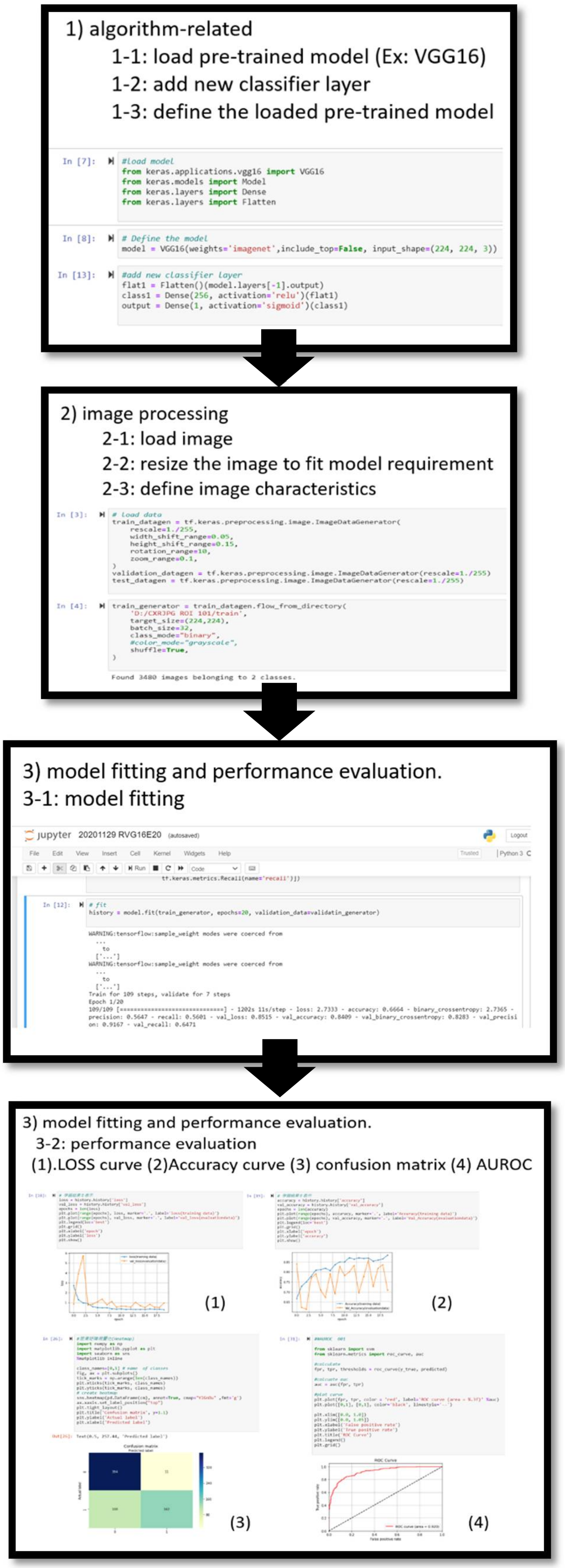

Figure S1. Flowchart showing Python code structure.

```
Model: "vgg16"
_________________________________________________________________
Layer (type)                 Output Shape              Param #
=================================================================
input_1 (InputLayer)         (None, 224, 224, 3)       0
_________________________________________________________________
block1_conv1 (Conv2D)        (None, 224, 224, 64)      1792
_________________________________________________________________
block1_conv2 (Conv2D)        (None, 224, 224, 64)      36928
_________________________________________________________________
block1_pool (MaxPooling2D)   (None, 112, 112, 64)      0
_________________________________________________________________
block2_conv1 (Conv2D)        (None, 112, 112, 128)     73856
_________________________________________________________________
block2_conv2 (Conv2D)        (None, 112, 112, 128)     147584
_________________________________________________________________
block2_pool (MaxPooling2D)   (None, 56, 56, 128)       0
_________________________________________________________________
block3_conv1 (Conv2D)        (None, 56, 56, 256)       295168
_________________________________________________________________
block3_conv2 (Conv2D)        (None, 56, 56, 256)       590080
_________________________________________________________________
block3_conv3 (Conv2D)        (None, 56, 56, 256)       590080
_________________________________________________________________
block3_pool (MaxPooling2D)   (None, 28, 28, 256)       0
_________________________________________________________________
block4_conv1 (Conv2D)        (None, 28, 28, 512)       1180160
_________________________________________________________________
block4_conv2 (Conv2D)        (None, 28, 28, 512)       2359808
_________________________________________________________________
block4_conv3 (Conv2D)        (None, 28, 28, 512)       2359808
_________________________________________________________________
block4_pool (MaxPooling2D)   (None, 14, 14, 512)       0
_________________________________________________________________
block5_conv1 (Conv2D)        (None, 14, 14, 512)       2359808
_________________________________________________________________
block5_conv2 (Conv2D)        (None, 14, 14, 512)       2359808
_________________________________________________________________
block5_conv3 (Conv2D)        (None, 14, 14, 512)       2359808
_________________________________________________________________
block5_pool (MaxPooling2D)   (None, 7, 7, 512)         0
=================================================================
Total params: 14,714,688
Trainable params: 14,714,688
Non-trainable params: 0
_________________________________________________________________
```

Figure S2. Summary of VGG16 model developed in this study.

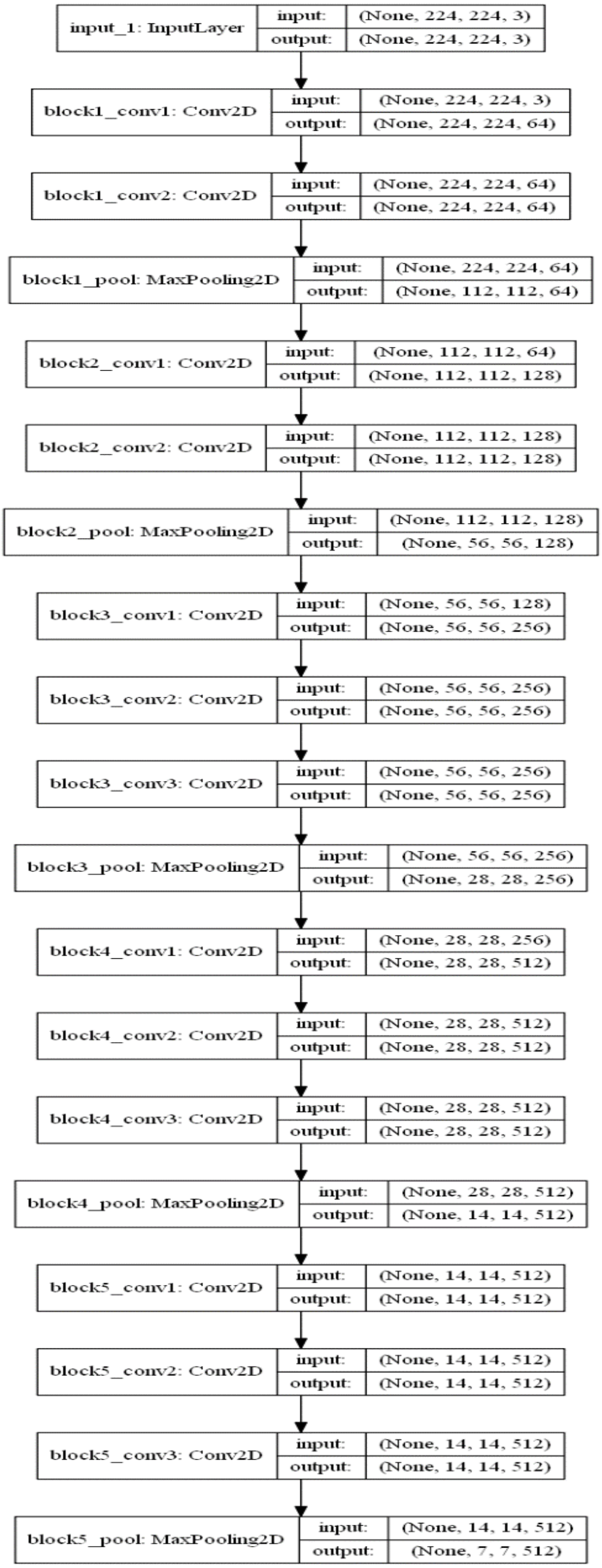

Figure S3. Model architecture of VGG16 model developed in this study.
